# 3-Benzyl-6-butyl-5-propyl-3*H*-1,2,3-triazolo[4,5-*d*]pyrimidin-7(6*H*)-one

**DOI:** 10.1107/S1600536810045575

**Published:** 2010-11-13

**Authors:** Xiao-Hua Zeng, Shou-Heng Deng, Hong-Mei Wang, Ai-Hua Zheng, Ping Chen

**Affiliations:** a Institute of Medicinal Chemistry, Hubei Medical Univesity, Shiyan 442000, People’s Republic of China; b Center of Oncology, People’s Hospital affiliated with Hubei Medical University, Shiyan 442000, People’s Republic of China

## Abstract

In the title compound, C_18_H_23_N_5_O_2_, the triazolopyrimidine ring system is essentially planar, with a maximum displacement of 0.032 (2) Å, and forms a dihedral angle of 87.59 (15)° with the phenyl ring. In the crystal, mol­ecules are linked by inter­molecular C—H⋯O hydrogen bonds and C—H⋯π inter­actions into chains parallel to the *c* axis.

## Related literature

For the biological activity of 8-aza­guanine derivatives, see: Roblin *et al.* (1945 ▶); Ding *et al.* (2004 ▶); Mitchell *et al.* (1950 ▶); Levine *et al.* (1963 ▶); Montgomery *et al.* (1962 ▶); Yamamoto *et al.* (1967 ▶); Bariana (1971 ▶); Holland *et al.* (1975 ▶); Zeng *et al.* (2010 ▶). For related structures, see: Ferguson *et al.* (1998 ▶); Li *et al.* (2004 ▶); Zhao, Xie *et al.* (2005 ▶); Zhao, Hu *et al.* (2005 ▶); Zhao, Wang & Ding (2005 ▶); Chen & Shi (2006 ▶); Maldonado *et al.* (2006 ▶); Xiao & Shi (2007 ▶); Wang *et al.* (2006 ▶, 2008 ▶); Zeng *et al.* (2006 ▶, 2009 ▶).
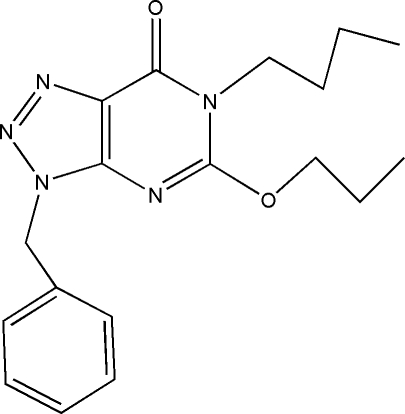


## Experimental

### 

#### Crystal data

C_18_H_23_N_5_O_2_
*M* *_r_* = 341.41
Orthorhombic, 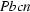

*a* = 28.328 (6) Å
*b* = 14.818 (3) Å
*c* = 8.7995 (16) Å
*V* = 3693.7 (12) Å^3^
*Z* = 8
Mo *K*α radiation
μ = 0.08 mm^−1^
*T* = 298 K
0.19 × 0.15 × 0.10 mm

#### Data collection

Bruker SMART CCD area-detector diffractometer
Absorption correction: multi-scan (*SADABS*; Sheldrick, 1996 ▶) *T*_min_ = 0.984, *T*_max_ = 0.992
18673 measured reflections
3346 independent reflections
2842 reflections with *I* > 2σ(*I*)
*R*_int_ = 0.041

#### Refinement

*R*[*F* ^2^ > 2σ(*F* ^2^)] = 0.080
*wR*(*F* ^2^) = 0.173
*S* = 1.21
3346 reflections
228 parameters
H-atom parameters constrained
Δρ_max_ = 0.30 e Å^−3^
Δρ_min_ = −0.15 e Å^−3^

### 

Data collection: *SMART* (Bruker, 2001 ▶); cell refinement: *SAINT* (Bruker, 2001 ▶); data reduction: *SAINT*; program(s) used to solve structure: *SHELXS97* (Sheldrick, 2008 ▶); program(s) used to refine structure: *SHELXL97* (Sheldrick, 2008 ▶); molecular graphics: *ORTEP-3 for Windows* (Farrugia, 1999 ▶) and *PLATON* (Spek, 2009) ▶; software used to prepare material for publication: *SHELXL97*.

## Supplementary Material

Crystal structure: contains datablocks global, I. DOI: 10.1107/S1600536810045575/rz2509sup1.cif

Structure factors: contains datablocks I. DOI: 10.1107/S1600536810045575/rz2509Isup2.hkl

Additional supplementary materials:  crystallographic information; 3D view; checkCIF report

## Figures and Tables

**Table 1:** Hydrogen-bond geometry (Å, °) *Cg*1 is the centroid of the the C1–C6 ring.

*D*—H⋯*A*	*D*—H	H⋯*A*	*D*⋯*A*	*D*—H⋯*A*
C2—H2⋯O1^i^	0.93	2.43	3.259 (4)	148
C15—H15*B*⋯*Cg*1^i^	0.97	2.94	3.711 (3)	137

Symmetry code: (i) 
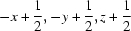
.

**Fig. 1.**
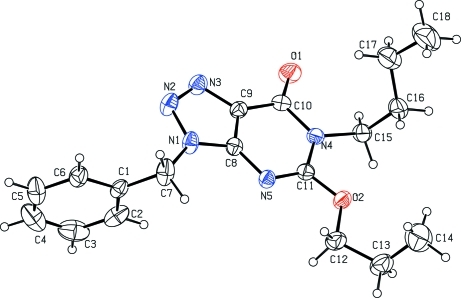
The molecular structure of the title compound showing the atom-labeling scheme. Displacement ellipsoids are drawn at the 50% probability level. H-atoms are represented by circles of arbitrary size.

## supplementary crystallographic information

### Comment

The derivatives of heterocycles containing the 8-azaguanine system, which are well known bioisosteres of guanine, are of great importance because of their remarkable biological properties. Some of these activities include antimicrobial or antifungal activities (Roblin *et al.*, 1945; Ding *et al.*, 2004; Zeng *et al.*, 2010), encephaloma cell inhibitor activity (Mitchell *et al.*, 1950; Levine *et al.*, 1963), antileukemie activity (Montgomery *et al.*, 1962), hypersusceptibility inhibitor activity and acesodyne activity (Yamamoto *et al.*, 1967; Bariana, 1971; Holland *et al.*, 1975).

In recent years, Ding's group has been engaged in the preparation of derivatives of 8-azaguanine *via* aza-Wittig reaction of β-ethoxycarbonyl iminophosphoranes with aromatic isocyanates (Zhao, Xie *et al.*, 2005). As a continuation of our research for new biologically active heterocycles, the title compound was obtained from β-ethoxycarbonyl iminophosphorane with alphalic isocyanate, and structurally characterized in this context.

In the title compound (Fig. 1), bond lengths and angles within the triazolopyrimidinone system are in good agreement with those observed for closely related structures (Zhao, Hu *et al.*, 2005; Zhao, Wang & Ding, 2005). As reported for related compounds (Ferguson *et al.*, 1998; Li *et al.*, 2004; Maldonado *et al.*, 2006; Zeng *et al.*, 2006, 2009; Wang *et al.*, 2006, 2008; Xiao & Shi, 2007; Chen & Shi, 2006), the triazolopyrimidine ring system is essentially planar, with a maximum displacement of 0.032 (2) Å for atom N4, and forms dihedral angles of 87.59 (15)° with the C1–C6 phenyl ring. In the crystal packing, molecules are linked by intermolecular C—H···O hydrogen bonds and C—H···π interactions (Table 1) into chains parallel to the *c* axis.

### Experimental

To the solution of carbodiimide prepared according to Zeng *et al.* (2006) in a mixed solvent (CH_2_Cl_2_/PrOH,1:4 *v*/*v*, 15 ml) was added a fresh prepared solution of Na/PrOH (0.1 g/2 ml). After stirring the reaction mixture for 6 h, the solvent was removed under reduced pressure and the residue was recrystallized from EtOH to give the title compound in 75% yield (m. p. 464 K). Elemental analysis: calculated for C_14_H_15_N_5_O_2_: C, 63.32; H, 6.79; N, 20.51%. Found: C, 62.75; H, 6.98; N, 20.22%. Crystals suitable for X-ray diffraction study were obtained by recrystallization from EtOH and dichloromethane (1:3 *v*/*v*) at room temperature.

### Refinement

All H atoms were placed at calculated positions and treated as riding atoms, with C—H = 0.93–0.97 Å and *U*_iso_(H) = 1.2*U*_eq_(C) or 1.5*U*_eq_(C) for methyl H atoms.

### Crystal data

**Table:**

C_18_H_23_N_5_O_2_	*D*_x_ = 1.228 Mg m^−^^3^
*M**_r_* = 341.41	Melting point: 364 K
Orthorhombic, *P**b**c**n*	Mo *K*α radiation, λ = 0.71073 Å
Hall symbol: -P 2n 2ab	Cell parameters from 3955 reflections
*a* = 28.328 (6) Å	θ = 2.6–23.4°
*b* = 14.818 (3) Å	µ = 0.08 mm^−^^1^
*c* = 8.7995 (16) Å	*T* = 298 K
*V* = 3693.7 (12) Å^3^	Block, colourless
*Z* = 8	0.19 × 0.15 × 0.10 mm
*F*(000) = 1456	

### Data collection

**Table:**

Bruker SMART CCD area-detector diffractometer	3346 independent reflections
Radiation source: fine-focus sealed tube	2842 reflections with *I* > 2σ(*I*)
graphite	*R*_int_ = 0.041
φ and ω scans	θ_max_ = 25.3°, θ_min_ = 2.6°
Absorption correction: multi-scan (*SADABS*; Sheldrick, 1996)	*h* = −34→29
*T*_min_ = 0.984, *T*_max_ = 0.992	*k* = −17→17
18673 measured reflections	*l* = −10→7

### Refinement

**Table:**

Refinement on *F*^2^	Primary atom site location: structure-invariant direct methods
Least-squares matrix: full	Secondary atom site location: difference Fourier map
*R*[*F*^2^ > 2σ(*F*^2^)] = 0.080	Hydrogen site location: inferred from neighbouring sites
*wR*(*F*^2^) = 0.173	H-atom parameters constrained
*S* = 1.21	*w* = 1/[σ^2^(*F*_o_^2^) + (0.0532*P*)^2^ + 2.6669*P*] where *P* = (*F*_o_^2^ + 2*F*_c_^2^)/3
3346 reflections	(Δ/σ)_max_ < 0.001
228 parameters	Δρ_max_ = 0.30 e Å^−^^3^
0 restraints	Δρ_min_ = −0.15 e Å^−^^3^

### Special details

**Table:**

Geometry. All e.s.d.'s (except the e.s.d. in the dihedral angle between two l.s. planes) are estimated using the full covariance matrix. The cell e.s.d.'s are taken into account individually in the estimation of e.s.d.'s in distances, angles and torsion angles; correlations between e.s.d.'s in cell parameters are only used when they are defined by crystal symmetry. An approximate (isotropic) treatment of cell e.s.d.'s is used for estimating e.s.d.'s involving l.s. planes.
Refinement. Refinement of *F*^2^ against ALL reflections. The weighted *R*-factor *wR* and goodness of fit *S* are based on *F*^2^, conventional *R*-factors *R* are based on *F*, with *F* set to zero for negative *F*^2^. The threshold expression of *F*^2^ > σ(*F*^2^) is used only for calculating *R*-factors(gt) *etc*. and is not relevant to the choice of reflections for refinement. *R*-factors based on *F*^2^ are statistically about twice as large as those based on *F*, and *R*- factors based on ALL data will be even larger.

### Fractional atomic coordinates and isotropic or equivalent isotropic displacement parameters (Å^2^)

**Table:**

	*x*	*y*	*z*	*U*_iso_*/*U*_eq_	
C1	0.13547 (10)	0.44798 (18)	0.0088 (3)	0.0454 (7)	
C2	0.11879 (15)	0.3707 (2)	0.0781 (4)	0.0689 (10)	
H2	0.1388	0.3363	0.1387	0.083*	
C3	0.07250 (19)	0.3444 (3)	0.0577 (5)	0.0900 (14)	
H3	0.0613	0.2929	0.1062	0.108*	
C4	0.04300 (15)	0.3936 (4)	−0.0333 (6)	0.0967 (15)	
H4	0.0120	0.3752	−0.0487	0.116*	
C5	0.05947 (13)	0.4692 (3)	−0.1007 (5)	0.0814 (12)	
H5	0.0394	0.5033	−0.1616	0.098*	
C6	0.10505 (11)	0.4963 (2)	−0.0807 (4)	0.0581 (8)	
H6	0.1157	0.5485	−0.1286	0.070*	
C7	0.18567 (11)	0.4789 (2)	0.0285 (4)	0.0674 (10)	
H7A	0.1902	0.5352	−0.0259	0.081*	
H7B	0.1917	0.4902	0.1354	0.081*	
C8	0.25650 (9)	0.37249 (17)	0.0422 (3)	0.0413 (6)	
C9	0.27483 (10)	0.31471 (18)	−0.0642 (3)	0.0424 (6)	
C10	0.31511 (10)	0.26037 (18)	−0.0287 (3)	0.0443 (7)	
C11	0.30961 (10)	0.33995 (18)	0.2144 (3)	0.0420 (6)	
C12	0.30873 (12)	0.4007 (2)	0.4643 (3)	0.0628 (9)	
H12A	0.2781	0.3761	0.4915	0.075*	
H12B	0.3042	0.4616	0.4263	0.075*	
C13	0.34015 (14)	0.4018 (3)	0.5988 (4)	0.0742 (10)	
H13A	0.3245	0.4351	0.6792	0.089*	
H13B	0.3441	0.3402	0.6339	0.089*	
C14	0.38675 (18)	0.4409 (4)	0.5757 (6)	0.131 (2)	
H14A	0.4045	0.4035	0.5076	0.197*	
H14B	0.4029	0.4449	0.6714	0.197*	
H14C	0.3836	0.5002	0.5330	0.197*	
C15	0.37466 (10)	0.2295 (2)	0.1713 (3)	0.0516 (8)	
H15A	0.3775	0.1745	0.1123	0.062*	
H15B	0.3705	0.2127	0.2769	0.062*	
C16	0.42007 (11)	0.2843 (3)	0.1552 (4)	0.0685 (10)	
H16A	0.4163	0.3403	0.2108	0.082*	
H16B	0.4455	0.2509	0.2031	0.082*	
C17	0.43453 (13)	0.3064 (3)	−0.0027 (5)	0.0896 (13)	
H17A	0.4092	0.3391	−0.0525	0.108*	
H17B	0.4397	0.2509	−0.0586	0.108*	
C18	0.47925 (15)	0.3631 (4)	−0.0072 (7)	0.1249 (19)	
H18A	0.4741	0.4185	0.0467	0.187*	
H18B	0.4873	0.3762	−0.1109	0.187*	
H18C	0.5046	0.3304	0.0396	0.187*	
N1	0.21950 (8)	0.41208 (15)	−0.0277 (3)	0.0508 (6)	
N2	0.21576 (10)	0.37955 (17)	−0.1729 (3)	0.0577 (7)	
N3	0.24886 (9)	0.32053 (17)	−0.1946 (3)	0.0537 (7)	
N4	0.33234 (8)	0.28021 (15)	0.1193 (2)	0.0417 (6)	
N5	0.27266 (8)	0.38784 (15)	0.1849 (3)	0.0430 (6)	
O1	0.33434 (8)	0.20431 (16)	−0.1081 (2)	0.0664 (7)	
O2	0.33084 (7)	0.34506 (14)	0.3490 (2)	0.0531 (6)	

### Atomic displacement parameters (Å^2^)

**Table:**

	*U*^11^	*U*^22^	*U*^33^	*U*^12^	*U*^13^	*U*^23^
C1	0.0575 (18)	0.0412 (15)	0.0375 (15)	0.0103 (13)	−0.0086 (13)	−0.0097 (12)
C2	0.108 (3)	0.053 (2)	0.0456 (18)	0.009 (2)	−0.0091 (19)	−0.0024 (15)
C3	0.115 (4)	0.068 (3)	0.087 (3)	−0.027 (3)	0.036 (3)	−0.010 (2)
C4	0.058 (2)	0.099 (3)	0.133 (4)	−0.009 (2)	0.016 (3)	−0.038 (3)
C5	0.055 (2)	0.085 (3)	0.105 (3)	0.015 (2)	−0.020 (2)	−0.006 (2)
C6	0.0590 (19)	0.0582 (18)	0.0572 (19)	0.0091 (16)	−0.0092 (16)	0.0052 (15)
C7	0.066 (2)	0.0520 (19)	0.085 (2)	0.0146 (16)	−0.0294 (19)	−0.0257 (18)
C8	0.0412 (15)	0.0374 (14)	0.0452 (16)	−0.0057 (12)	−0.0025 (12)	−0.0010 (12)
C9	0.0454 (16)	0.0429 (15)	0.0390 (15)	−0.0034 (13)	−0.0016 (12)	−0.0010 (12)
C10	0.0472 (16)	0.0444 (15)	0.0413 (16)	−0.0007 (13)	0.0087 (13)	−0.0003 (13)
C11	0.0429 (15)	0.0421 (14)	0.0409 (15)	−0.0011 (13)	0.0029 (12)	0.0021 (12)
C12	0.068 (2)	0.076 (2)	0.0448 (18)	0.0197 (18)	0.0019 (15)	−0.0107 (16)
C13	0.089 (3)	0.081 (2)	0.053 (2)	0.009 (2)	−0.0058 (19)	−0.0148 (18)
C14	0.098 (4)	0.184 (6)	0.112 (4)	−0.033 (4)	−0.004 (3)	−0.051 (4)
C15	0.0522 (18)	0.0529 (17)	0.0495 (17)	0.0156 (14)	0.0040 (14)	0.0069 (14)
C16	0.0506 (19)	0.086 (2)	0.069 (2)	0.0180 (18)	0.0039 (16)	0.0131 (19)
C17	0.067 (2)	0.113 (3)	0.089 (3)	0.002 (2)	0.017 (2)	0.018 (3)
C18	0.079 (3)	0.148 (5)	0.148 (5)	−0.014 (3)	0.030 (3)	0.038 (4)
N1	0.0492 (14)	0.0432 (13)	0.0601 (16)	0.0056 (11)	−0.0176 (12)	−0.0115 (12)
N2	0.0653 (17)	0.0535 (15)	0.0543 (16)	0.0024 (14)	−0.0215 (13)	−0.0074 (13)
N3	0.0568 (15)	0.0554 (15)	0.0489 (15)	0.0000 (13)	−0.0086 (12)	−0.0075 (12)
N4	0.0414 (13)	0.0439 (12)	0.0397 (12)	0.0042 (10)	0.0042 (10)	0.0027 (10)
N5	0.0430 (13)	0.0439 (13)	0.0423 (13)	0.0047 (11)	−0.0054 (10)	−0.0054 (10)
O1	0.0752 (15)	0.0695 (14)	0.0544 (13)	0.0225 (12)	0.0036 (11)	−0.0173 (12)
O2	0.0539 (12)	0.0661 (13)	0.0393 (11)	0.0165 (10)	−0.0054 (9)	−0.0067 (9)

### Geometric parameters (Å, °)

**Table:**

C1—C6	1.370 (4)	C12—O2	1.450 (3)
C1—C2	1.381 (4)	C12—C13	1.481 (5)
C1—C7	1.504 (4)	C12—H12A	0.9700
C2—C3	1.380 (6)	C12—H12B	0.9700
C2—H2	0.9300	C13—C14	1.456 (6)
C3—C4	1.368 (6)	C13—H13A	0.9700
C3—H3	0.9300	C13—H13B	0.9700
C4—C5	1.350 (6)	C14—H14A	0.9600
C4—H4	0.9300	C14—H14B	0.9600
C5—C6	1.364 (5)	C14—H14C	0.9600
C5—H5	0.9300	C15—N4	1.486 (3)
C6—H6	0.9300	C15—C16	1.528 (4)
C7—N1	1.464 (4)	C15—H15A	0.9700
C7—H7A	0.9700	C15—H15B	0.9700
C7—H7B	0.9700	C16—C17	1.485 (5)
C8—N1	1.350 (3)	C16—H16A	0.9700
C8—N5	1.356 (3)	C16—H16B	0.9700
C8—C9	1.371 (4)	C17—C18	1.521 (6)
C9—N3	1.366 (4)	C17—H17A	0.9700
C9—C10	1.431 (4)	C17—H17B	0.9700
C10—O1	1.215 (3)	C18—H18A	0.9600
C10—N4	1.421 (3)	C18—H18B	0.9600
C11—N5	1.291 (3)	C18—H18C	0.9600
C11—O2	1.331 (3)	N1—N2	1.369 (3)
C11—N4	1.378 (3)	N2—N3	1.296 (3)
			
C6—C1—C2	118.2 (3)	C12—C13—H13A	108.3
C6—C1—C7	120.1 (3)	C14—C13—H13B	108.3
C2—C1—C7	121.7 (3)	C12—C13—H13B	108.3
C3—C2—C1	120.1 (4)	H13A—C13—H13B	107.4
C3—C2—H2	119.9	C13—C14—H14A	109.5
C1—C2—H2	119.9	C13—C14—H14B	109.5
C4—C3—C2	120.4 (4)	H14A—C14—H14B	109.5
C4—C3—H3	119.8	C13—C14—H14C	109.5
C2—C3—H3	119.8	H14A—C14—H14C	109.5
C5—C4—C3	119.2 (4)	H14B—C14—H14C	109.5
C5—C4—H4	120.4	N4—C15—C16	112.5 (2)
C3—C4—H4	120.4	N4—C15—H15A	109.1
C4—C5—C6	121.0 (4)	C16—C15—H15A	109.1
C4—C5—H5	119.5	N4—C15—H15B	109.1
C6—C5—H5	119.5	C16—C15—H15B	109.1
C5—C6—C1	121.0 (3)	H15A—C15—H15B	107.8
C5—C6—H6	119.5	C17—C16—C15	115.9 (3)
C1—C6—H6	119.5	C17—C16—H16A	108.3
N1—C7—C1	111.9 (2)	C15—C16—H16A	108.3
N1—C7—H7A	109.2	C17—C16—H16B	108.3
C1—C7—H7A	109.2	C15—C16—H16B	108.3
N1—C7—H7B	109.2	H16A—C16—H16B	107.4
C1—C7—H7B	109.2	C16—C17—C18	112.1 (4)
H7A—C7—H7B	107.9	C16—C17—H17A	109.2
N1—C8—N5	127.7 (2)	C18—C17—H17A	109.2
N1—C8—C9	104.8 (2)	C16—C17—H17B	109.2
N5—C8—C9	127.5 (3)	C18—C17—H17B	109.2
N3—C9—C8	109.3 (2)	H17A—C17—H17B	107.9
N3—C9—C10	130.4 (3)	C17—C18—H18A	109.5
C8—C9—C10	120.3 (2)	C17—C18—H18B	109.5
O1—C10—N4	121.0 (3)	H18A—C18—H18B	109.5
O1—C10—C9	128.1 (3)	C17—C18—H18C	109.5
N4—C10—C9	110.9 (2)	H18A—C18—H18C	109.5
N5—C11—O2	120.9 (2)	H18B—C18—H18C	109.5
N5—C11—N4	127.6 (2)	C8—N1—N2	109.4 (2)
O2—C11—N4	111.5 (2)	C8—N1—C7	130.4 (3)
O2—C12—C13	107.8 (3)	N2—N1—C7	120.2 (2)
O2—C12—H12A	110.1	N3—N2—N1	108.6 (2)
C13—C12—H12A	110.1	N2—N3—C9	108.0 (2)
O2—C12—H12B	110.1	C11—N4—C10	121.9 (2)
C13—C12—H12B	110.1	C11—N4—C15	121.0 (2)
H12A—C12—H12B	108.5	C10—N4—C15	117.0 (2)
C14—C13—C12	116.0 (4)	C11—N5—C8	111.6 (2)
C14—C13—H13A	108.3	C11—O2—C12	117.4 (2)
			
C6—C1—C2—C3	−0.7 (5)	C1—C7—N1—C8	−125.7 (3)
C7—C1—C2—C3	179.9 (3)	C1—C7—N1—N2	53.6 (4)
C1—C2—C3—C4	1.2 (6)	C8—N1—N2—N3	0.7 (3)
C2—C3—C4—C5	−1.3 (6)	C7—N1—N2—N3	−178.8 (3)
C3—C4—C5—C6	0.8 (7)	N1—N2—N3—C9	−0.6 (3)
C4—C5—C6—C1	−0.3 (6)	C8—C9—N3—N2	0.4 (3)
C2—C1—C6—C5	0.2 (5)	C10—C9—N3—N2	−179.7 (3)
C7—C1—C6—C5	179.7 (3)	N5—C11—N4—C10	−3.3 (4)
C6—C1—C7—N1	−118.6 (3)	O2—C11—N4—C10	177.3 (2)
C2—C1—C7—N1	60.9 (4)	N5—C11—N4—C15	−179.7 (3)
N1—C8—C9—N3	0.0 (3)	O2—C11—N4—C15	0.9 (3)
N5—C8—C9—N3	−178.7 (3)	O1—C10—N4—C11	−176.4 (3)
N1—C8—C9—C10	−179.9 (2)	C9—C10—N4—C11	4.2 (3)
N5—C8—C9—C10	1.4 (4)	O1—C10—N4—C15	0.1 (4)
N3—C9—C10—O1	−2.5 (5)	C9—C10—N4—C15	−179.2 (2)
C8—C9—C10—O1	177.3 (3)	C16—C15—N4—C11	−83.6 (3)
N3—C9—C10—N4	176.8 (3)	C16—C15—N4—C10	99.8 (3)
C8—C9—C10—N4	−3.4 (3)	O2—C11—N5—C8	−179.8 (2)
O2—C12—C13—C14	62.4 (5)	N4—C11—N5—C8	0.8 (4)
N4—C15—C16—C17	−65.6 (4)	N1—C8—N5—C11	−178.3 (3)
C15—C16—C17—C18	178.4 (3)	C9—C8—N5—C11	0.1 (4)
N5—C8—N1—N2	178.3 (3)	N5—C11—O2—C12	4.8 (4)
C9—C8—N1—N2	−0.4 (3)	N4—C11—O2—C12	−175.8 (2)
N5—C8—N1—C7	−2.4 (5)	C13—C12—O2—C11	−175.7 (3)
C9—C8—N1—C7	179.0 (3)		

### Hydrogen-bond geometry (Å, °)

**Table:**

Cg1 is the centroid of the the C1–C6 ring.

**Table:**

*D*—H···*A*	*D*—H	H···*A*	*D*···*A*	*D*—H···*A*
C2—H2···O1^i^	0.93	2.43	3.259 (4)	148
C15—H15B···Cg1^i^	0.97	2.94	3.711 (3)	137

Symmetry codes: (i) −*x*+1/2, −*y*+1/2, *z*+1/2.
